# Supplementary figures and images for: Adaptation Dynamics in Densely Clustered Chemoreceptors
Source: PLoS Comput Biol. 2013 Sep 19;9(9):e1003230. doi: 10.1371/journal.pcbi.1003230 (PMC3777915; doi:10.1371/journal.pcbi.1003230)

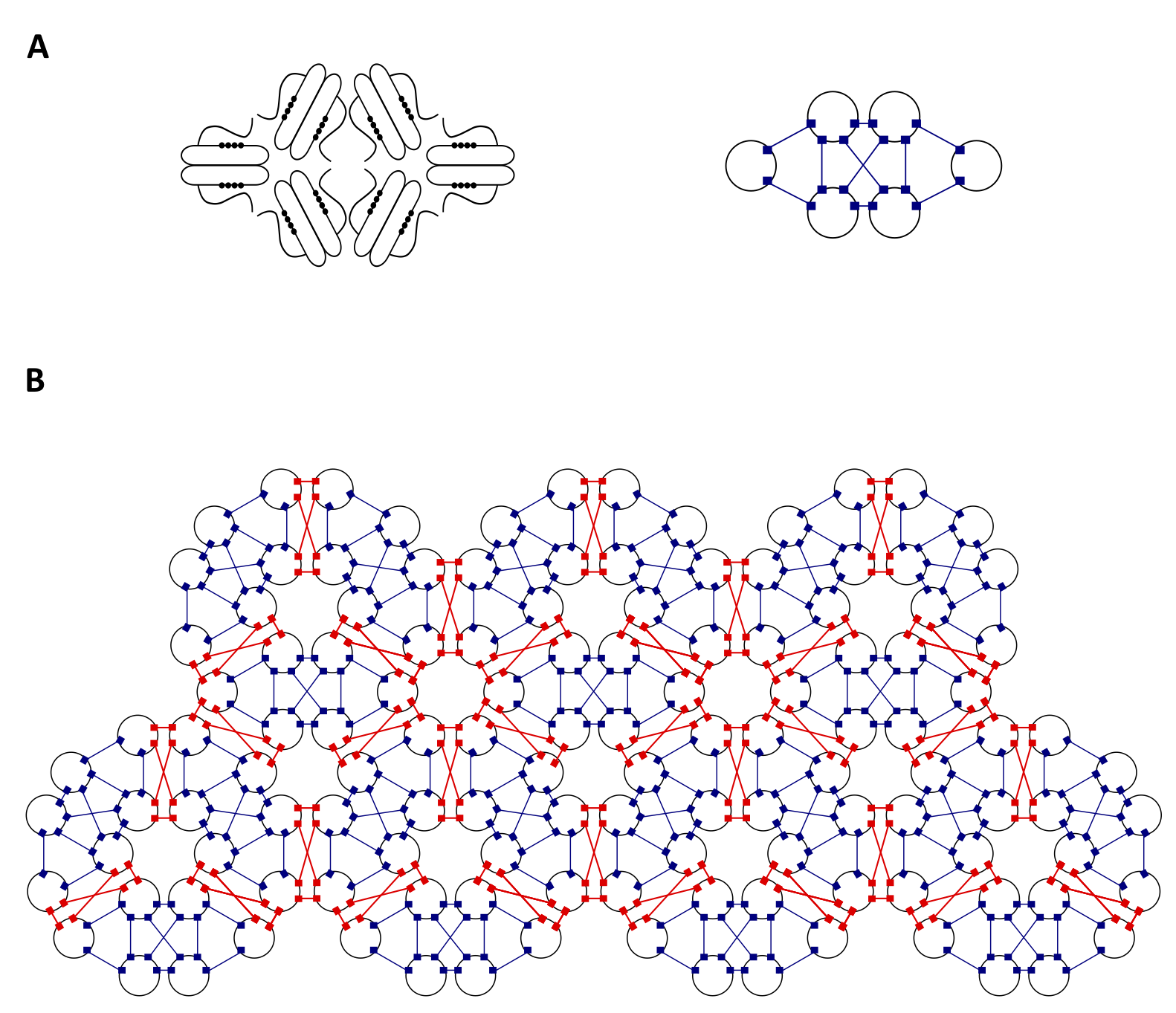

Supplement: Figure S1 — Structuring the chemoreceptor lattice in NFsim. (A) A MWC signaling complex consisting of two trimers of dimers (left) is specified by enumerating bonds (right, blue) between a dimer and all of its neighbors within the complex. (B) The hexagonal lattice is then structured by enumerating bonds between a given dimer and all of its neighbors in other signaling complexes (red). The pictured lattice consists of 21 MWC complexes. All interior dimers have six neighbors. The basic unit of the lattice is the hexagon consisting of three signaling complexes. We model lattices of equal length and width, as specified in terms of this basic hexagonal unit. (TIFF) [file pcbi.1003230.s001.tiff]

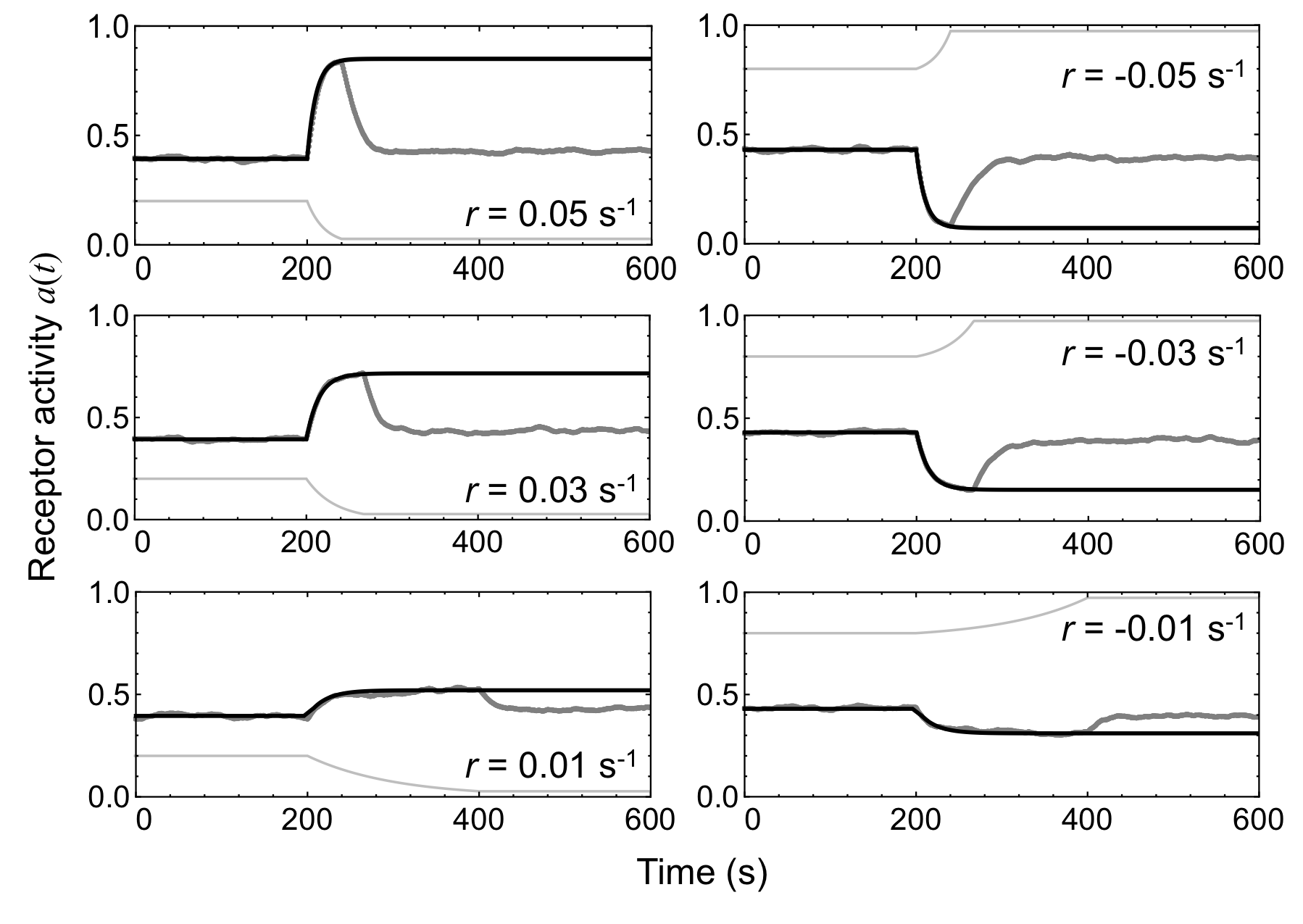

Supplement: Figure S2 — Response of the numerical model M1 to time-varying exponential ramps of chemoattractant. We presented the simulated cells with exponential ramps of methyl-aspartate (light gray, plotted in arbitrary units) of rate r (shown in each panel) and averaged the response in receptor activity over ten trials (dark gray). For each ramp, receptor activity approached a steady-state value during stimulus, determined by exponential fits (black) to a(t) and plotted in Fig. 2A of the main text. Following a recent experiment [43], the methyl-aspartate concentration ranged between 0.084 and 0.62 mM. (TIFF) [file pcbi.1003230.s002.tiff]

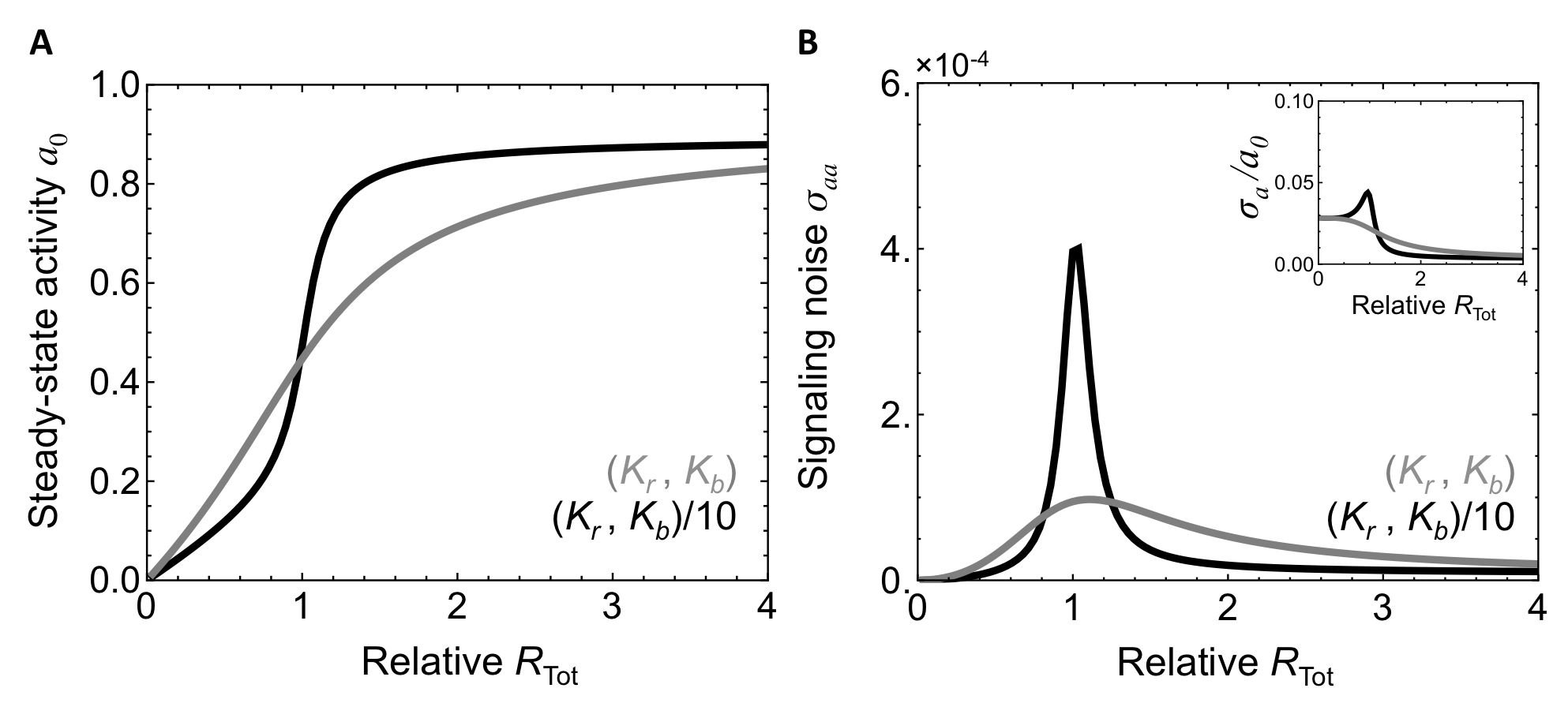

Supplement: Figure S3 — Fluctuations in the analytical model with no enzyme localization. The noise level within a narrow range of CheR values increases as the dependence of the steady- state activity on CheR count becomes steeper. (A) Steady state activity a 0 as a function of normalized CheR count for the parameters used in Fig. 4 (gray) and with Michaelis-Menten constants Kr and Kb reduced by a factor of 10 (black). The latter curve exhibits an extreme dependence on variations in CheR count. (B) Variance σaa and relative noise σa/a0 (inset) in activity at the steady state as a function of normalized CheR count for original (gray) and reduced Kr and Kb (black). Reducing Kr and Kb increases the relative noise level to nearly 5%. (TIFF) [file pcbi.1003230.s003.tiff]

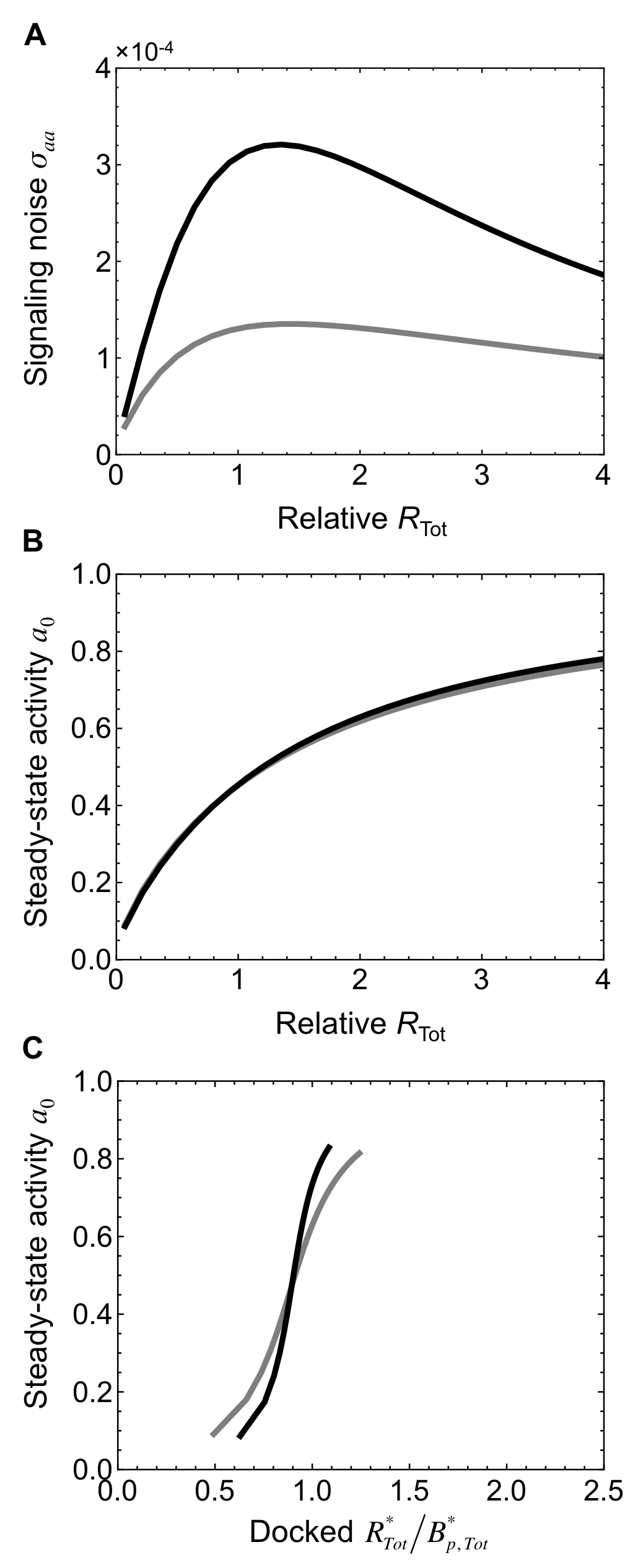

Supplement: Figure S4 — Increasing the distributivity of methylation in the detailed analytical model (Text S1) increases noise and the affinity of localized enzymes for the receptor substrate. (A) Variance σaa in overall activity as a function of total CheR count for fully processive methylation, β = 0 (gray), and more distributive methylation, β = 20 s−1 (black) (B) The steady-state activity a 0 as a function of total CheR is similar for both β = 0 (gray) and β = 20 s−1 (black). (C) Steady-state activity a 0 versus localized CheR/CheB-P, , is much steeper in the more distributive model with β = 20 s−1 (black) than β = 0 (gray). (TIFF) [file pcbi.1003230.s004.tiff]

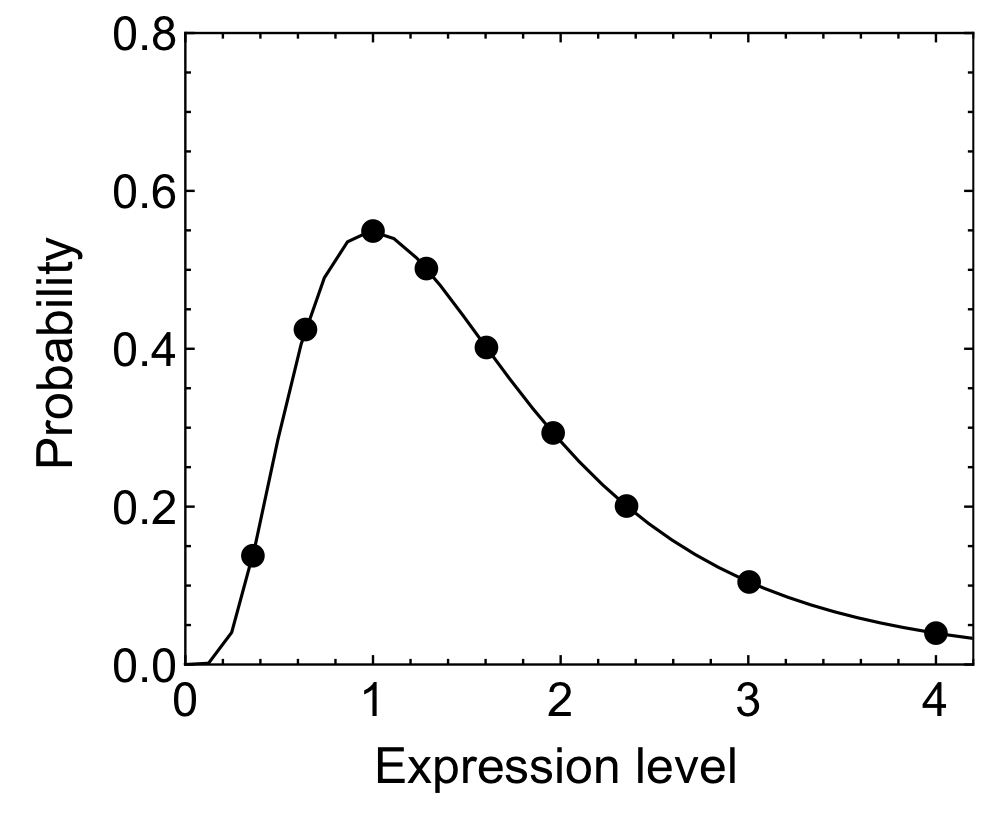

Supplement: Figure S5 — Estimated distribution of overall chemotaxis protein expression levels in a wild-type population relative to the most common expression level. We sampled representative cells (points) from a population in which the ratio CheR/CheB/chemoreceptors is maintained while the overall expression level follows a log-normal distribution. Signaling noise levels for these representative cells are shown in Fig. 3A of the main text. (TIFF) [file pcbi.1003230.s005.tiff]

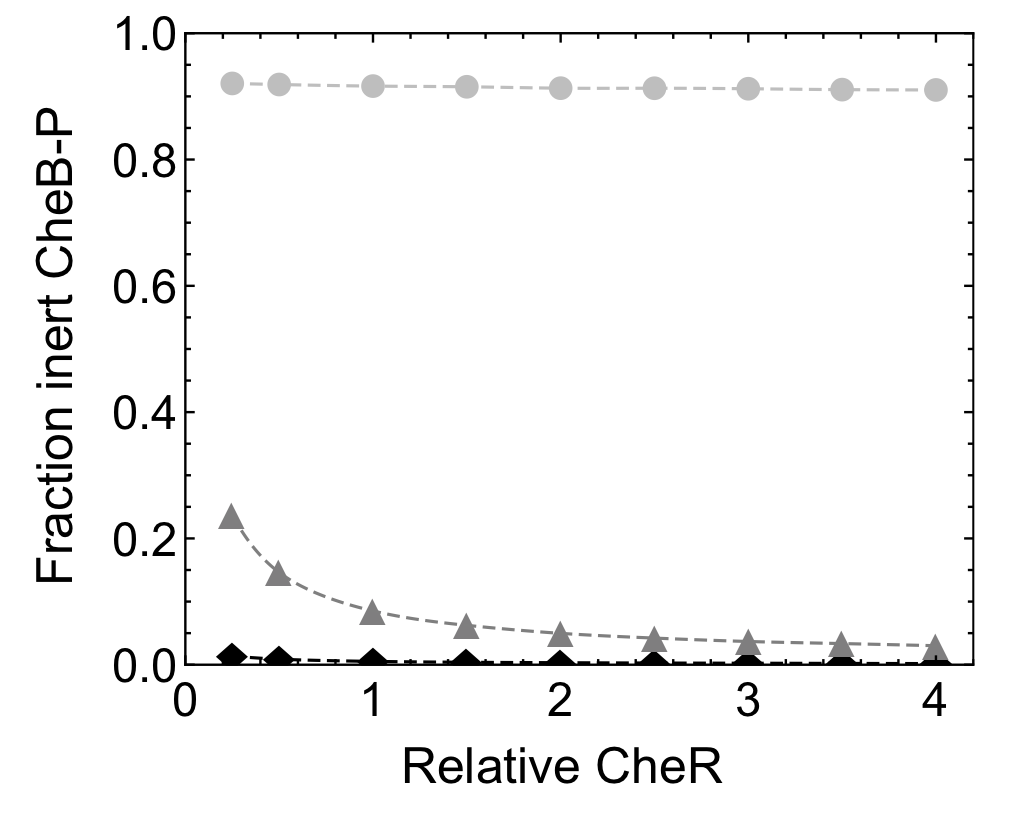

Supplement: Figure S6 — Mean fraction of “inert” CheB-P tethered within fully demethylated assistance neighborhoods (for models M1, black, and M3, light gray) or with fully demethylated receptor dimers (M2, dark gray) versus total CheR. These enzymes may bind the modification sites of receptors but will be unable to demethylate once bound. These enzymes are unable to affect the activity of the receptor cluster and are therefore not counted when calculating the ratio of localized CheR to CheB-P for Fig. 4. Since very few receptors are fully methylated, the number of inert, localized CheR is negligible (<1) for all models. This situation arises because MWC signaling complexes are highly active even at low methylation levels: in the absence of stimulus, a = 0.5 for m = 6 (out of 48) and a ∼ for m = 14. Consequently, many receptor dimers are fully demethylated even for cases in which the average receptor activity is high. In contrast, full methylated dimers are rare. (TIFF) [file pcbi.1003230.s006.tiff]

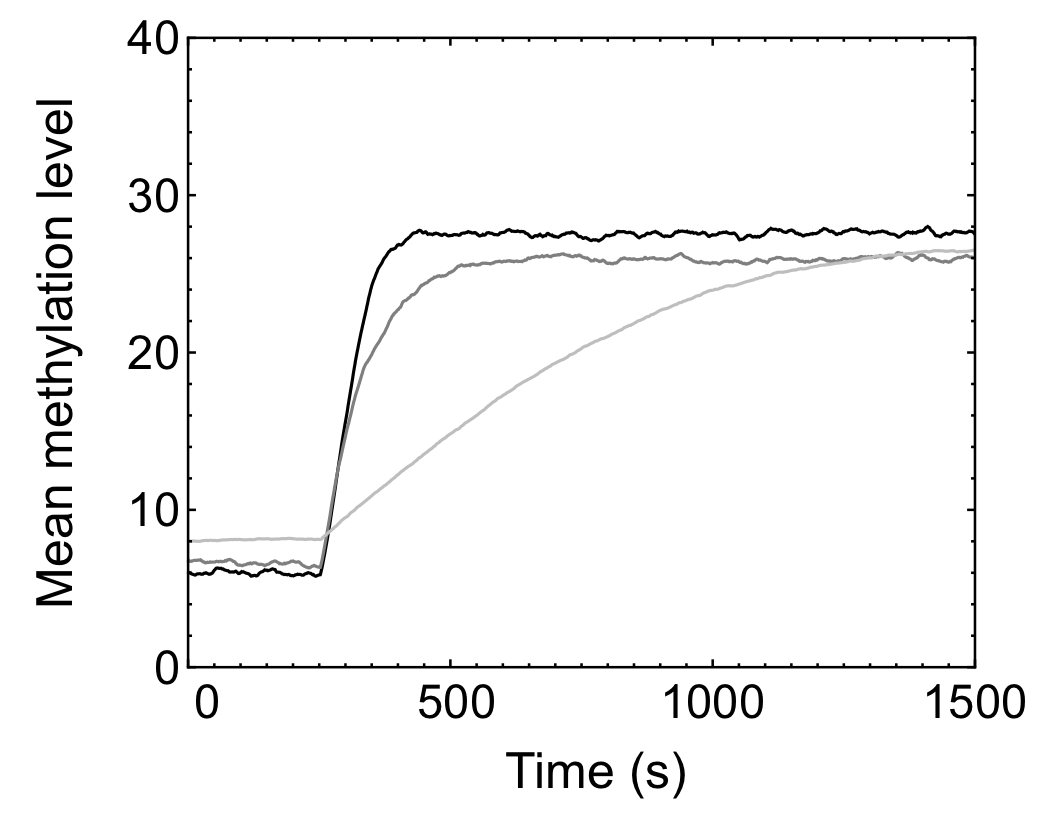

Supplement: Figure S7 — Average methylation level per MWC complex as a function of time for numerical models M1 (black), M2 (light gray), and M3 (dark gray) during the simulations shown in Fig. 2B (lower panel) of the main text. A step stimulus of 1 mM MeAsp was presented at 200 s. The most distributive model M1 displays the highest methylation rate during the adaptation process. (TIFF) [file pcbi.1003230.s007.tiff]
