# Supplementary material for: Adaptation Dynamics in Densely Clustered Chemoreceptors
Source: PLoS Comput Biol. 2013 Sep 19;9(9):e1003230. doi: 10.1371/journal.pcbi.1003230 (PMC3777915; doi:10.1371/journal.pcbi.1003230)
Supplement: Table S1 — Parameter names and values common to all models. (PDF) [file pcbi.1003230.s008.pdf]

Total number of receptor monomers (base)	$T_{Tot,0}$	14400
Total number of CheR (base)	$R_{Tot,0}$	140
Total number of CheB (base)	$B_{Tot,0}$	240
Number of receptor dimers per MWC cluster	N	6
Basal free energy difference, active and inactive cluster	ε_0	6 (units of k_BT)
Free energy change per added methyl group	ε_1	1 (units of k_BT)
MeAsp dissociation constant, inactive Tar receptor	K	0.0182 mM
MeAsp dissociation constant, active Tar receptor	K^*	3 mM
