# Supplementary material for: Adaptation Dynamics in Densely Clustered Chemoreceptors
Source: PLoS Comput Biol. 2013 Sep 19;9(9):e1003230. doi: 10.1371/journal.pcbi.1003230 (PMC3777915; doi:10.1371/journal.pcbi.1003230)
Supplement: Table S2 — Parameter values for stochastic simulation of model M1 with enzyme localization. Rates are designated as in Fig. 1B with an r or b subscript to denote rates of CheR and CheB reactions. (PDF) [file pcbi.1003230.s009.pdf]

Bulk CheR binding to tether	a_r^t	1/14.7 $s^{-1}/T_{Tot,0}$
Bulk CheR binding to modification site	a_r^m	0.0245 $s^{-1}/T_{Tot,0}$
Tethered CheR binding modification site, same receptor	a_{r*}^m	400 s^{-1}
CheR binding tether while attached to mod. site, same receptor	a_{r*}^t	1110 s^{-1}
Tethered CheR binding modification site, neighboring receptor	$a_{r*}^{m'}$	400 s^{-1}
CheR binding tether while attached to mod. site, neighboring rec.	$a_{r*}^{t'}$	1110 s^{-1}
CheR unbinding modification site	d_r^m	9.3 s^{-1}
CheR unbinding tether site	d_r^t	5 s^{-1}
CheR catalytic rate	k_r	2.7 s^{-1}
Bulk CheB-P binding to tether	a_b^t	1/16.3 $s^{-1}/T_{Tot,0}$
Bulk CheB-P binding to modification site	a_b^m	0.0245 $s^{-1}/T_{Tot,0}$
Tethered CheB-P binding modification site, same receptor	a_{b*}^m	400 s^{-1}
CheB-P binding tether while attached to mod. site	a_{b*}^t	1000 s^{-1}
Tethered CheB-P binding modification site, neighboring receptor	$a_{b*}^{m'}$	400 s^{-1}
CheB-P binding tether while attached to mod. site, neighboring rec.	$a_{b*}^{t'}$	1000 s^{-1}
CheB-P unbinding modification site	d_b^m	9 s^{-1}
CheB-P unbinding tether site	d_b^t	5 s^{-1}
CheB-P catalytic rate	k_b	3 s^{-1}
CheB phosphorylation rate	a_p	3 s^{-1}/ T_{Tot}
CheB-P dephosphorylation rate	d_p	0.37 s^{-1}
