# Supplementary material for: Adaptation Dynamics in Densely Clustered Chemoreceptors
Source: PLoS Comput Biol. 2013 Sep 19;9(9):e1003230. doi: 10.1371/journal.pcbi.1003230 (PMC3777915; doi:10.1371/journal.pcbi.1003230)
Supplement: Table S3 — Parameter values for stochastic simulation of the model B1 with no enzyme localization. (PDF) [file pcbi.1003230.s010.pdf]

<table>
<tr><td>CheR catalytic rate</td><td>k_r</td><td rowspan="4">$2N\ (0.03\ T_{Tot}/R_{Tot})\ s^{-1}$</td></tr>
<tr><td>CheB catalytic rate</td><td>k_b</td></tr>
<tr><td>CheR unbinding modification site</td><td>d_r</td></tr>
<tr><td>CheB unbinding modification site</td><td>d_b</td></tr>
<tr><td>CheR binding to modification site</td><td>a_r</td><td>$(k_r + d_r)/(0.43\ T_{Tot})$</td></tr>
<tr><td>CheB binding to modification site</td><td>a_b</td><td>$(k_b + d_b)/(0.3\ T_{Tot})$</td></tr>
</table>
