# Supplementary material for: Adaptation Dynamics in Densely Clustered Chemoreceptors
Source: PLoS Comput Biol. 2013 Sep 19;9(9):e1003230. doi: 10.1371/journal.pcbi.1003230 (PMC3777915; doi:10.1371/journal.pcbi.1003230)
Supplement: Table S4 — Parameter values for mean-field analytical model with enzyme localization. All values are derived from values of corresponding parameters in the numerical model M1 (Table S2). (PDF) [file pcbi.1003230.s011.pdf]

Bulk CheR binding to tether	a_r^t	1/14.7 $s^{-1}/T_{Tot,0}$
CheR unbinding tether	d_r^t	5 s^{-1}
Bulk CheB binding to tether	a_b^t	1/16.3 $s^{-1}/T_{Tot,0}$
CheB unbinding tether	d_b^t	5 s^{-1}
CheR catalytic rate	k_r	2.7 s^{-1}
CheB catalytic rate	k_b	3 s^{-1}
Tethered CheR modification site affinity	K_r	0.03
Tethered CheB modification site affinity	K_b	0.03
CheB phosphorylation rate	a_p	3 s^{-1}/ T_{Tot}
CheB-P dephosphorylation rate	d_p	0.37 s^{-1}
