# Supplementary material for: Adaptation Dynamics in Densely Clustered Chemoreceptors
Source: PLoS Comput Biol. 2013 Sep 19;9(9):e1003230. doi: 10.1371/journal.pcbi.1003230 (PMC3777915; doi:10.1371/journal.pcbi.1003230)
Supplement: Table S5 — Parameter values for analytical version of model B1 with no enzyme localization. (PDF) [file pcbi.1003230.s012.pdf]

CheR catalytic rate	k_r	$2N$ $(0.03\ T_{Tot}/R_{Tot})$ s^{-1}
CheB catalytic rate	k_b	$2N$ $(0.03\ T_{Tot}/B_{Tot})$ s^{-1}
Tethered CheR modification site affinity	K_r	0.43 T_{Tot}
Tethered CheB modification site affinity	K_b	0.3 T_{Tot}
