# Supplementary material for: Adaptation Dynamics in Densely Clustered Chemoreceptors
Source: PLoS Comput Biol. 2013 Sep 19;9(9):e1003230. doi: 10.1371/journal.pcbi.1003230 (PMC3777915; doi:10.1371/journal.pcbi.1003230)
Supplement: Table S6 — Changes in parameter values for the derived models M2, M3, and B2. (PDF) [file pcbi.1003230.s013.pdf]

| Model | Base model | Parameters changed from base model |
|---|---|---|
| **M2** | **M1** | $a_{r*}^{m'} = a_{r*}^{t'} = a_{b*}^{m'} = a_{b*}^{m'} = 0$ |
| **M3** | **M1** | $d_r^t = d_b^t = 0.25\ \mathrm{s}^{-1}$ |
| **B2** | **B1** | $a_r$, $a_b$ increased tenfold |
| **B2** (analytical) | **B1** (analytical) | $K_r$, $K_b$ increased tenfold |
