# Supplementary material for: Adaptation Dynamics in Densely Clustered Chemoreceptors
Source: PLoS Comput Biol. 2013 Sep 19;9(9):e1003230. doi: 10.1371/journal.pcbi.1003230 (PMC3777915; doi:10.1371/journal.pcbi.1003230)
Supplement: Table S7 — Number of unique dimers visited by localized enzymes per second for the numerical models. Higher rates indicate more distributive methylation. (PDF) [file pcbi.1003230.s014.pdf]

Model	**M1**	**M2**	**M3**
CheR sampling rate (dimers/s)	4.1	1.1	0.64
CheB-P sampling rate (dimers/s)	3.4	0.26	0.36
