# Supplementary material for: Adaptation Dynamics in Densely Clustered Chemoreceptors
Source: PLoS Comput Biol. 2013 Sep 19;9(9):e1003230. doi: 10.1371/journal.pcbi.1003230 (PMC3777915; doi:10.1371/journal.pcbi.1003230)
Supplement: Text S1 — Additional details regarding model derivations, implementations, and analysis. (PDF) [file pcbi.1003230.s015.pdf]

# Supporting Text S1

## Adaptation Dynamics in Densely Clustered Chemoreceptors

William Pontius[1,2], Michael W. Sneddon[2,3,4], Thierry Emonet[1,2]

[1]Department of Physics, Yale University, New Haven, CT, USA

[2]Department of Molecular, Cellular, and Developmental Biology, Yale University, New Haven, CT, USA

[3]Interdepartmental Program in Computational Biology and Bioinformatics, Yale University, New Haven, CT, USA

[4]Current address: Physical Biosciences Division, Lawrence Berkeley National Lab, Berkeley, CA, USA

---

### NFsim implementation of the receptor lattice model

NFsim [1] is a rule-based stochastic simulator of chemical reaction networks built on the BioNetGen language [2]. It is designed to efficiently simulate systems in which molecules may exist in large numbers of states, and in which these states affect the rates of the reactions in which molecules participate. To illustrate the problem, we consider the case of a bacterial chemoreceptor in a MWC signaling complex. The rate of CheR binding to the modification site depends on: (1) whether the receptor modification site is occupied (by CheR or CheB); (2) whether the enzyme active site is occupied (by CheR or CheB); (3) whether the enzyme is tethered to the receptor, tethered to a neighboring receptor, tethered to a non-neighboring receptor, or in the bulk; and (4) the methylation level of the signaling complex in which the receptor is located, which varies between 0 and 48 for a complex of six dimers. Accordingly, the reaction proceeds with a rate specific to each of the 3×3×4×49 = 1764 possible receptor-enzyme states. NFsim enables us to fully specify the above model with relatively few explicit reaction rules. Moreover, the speed of simulation in NFsim scales nearly independently of the number of possible states [1].

In the simulation, chemoreceptor dimers are specified by objects of the form `T(m,mc,as,teth,[loc],[hex])` in which `m` and `mc` denote the methylation level of the dimer (0 to 8) and the local MWC cluster (0 to 48), `as` and `teth` are binding sites representing the active site and tether respectively, and `[hex]` and `[loc]` are each a series of binding sites used to specify the organization of the receptor lattice. While NFsim does not support spatially resolved simulations, we can specify the neighbors of a given dimer by creating bonds between it and all of its neighboring dimers. Fig. S1A illustrates how a MWC cluster of six dimers is specified by creating bonds (blue lines) between the `[loc]` sites (blue squares) on each dimer. Fig. S1B illustrates 21 MWC clusters assembled into a hexagonal lattice by specifying bonds (red lines) between the `[hex]` sites (red squares) of neighboring dimers. All interior dimers are connected to six neighboring dimers. These bonds need not correspond to chemical bonds in the

actual system; here they are a feature of the simulation language that we use to specify the lattice organization.

To illustrate reaction rules, we consider the reaction in which a tethered CheR binds to the active site of a neighboring receptor. The corresponding rule is

```
R(as,teth!1).T(teth!1,hex!2).T%t(as,hex!2) ->

            R(as!3,teth!1).T(teth!1,hex!2).T%t(as!3,hex!2).
```

CheR is represented by the object R with two binding sites `as` and `teth`. The dot notation indicates that two objects are bound and the `!n` notation serves to label distinct bonds. `T%t` indicates that the reaction rate is a function of the state of the object `T`, referenced as `t` in the function argument. In this case, the reaction rate is a basal binding rate times one minus the activity of `t`, calculated in the simulation by evaluating Eq. (13) at the methylation level `mc` of `t`. Functionally defined rate laws are a key feature of NFsim that in this illustrative case save us from having to define a separate reaction for each value of `mc` and `m`.

For simplicity, we have assumed that each dimer has one modification site and one tether site. This simplification should not affect the results significantly since the number of receptors greatly exceeds the number of adaptation enzymes [3]. The simulation described in this section and in the main text was used for models **M1**, **M2**, and **M3**. Parameters for these models are given in Tables S1, S2, S6 and are discussed below.

**Implementation of models with no enzyme localization**

The models **B1** and **B2** were also simulated using NFsim. MWC signaling complexes were modeled as objects with a methylation level ranging from 0 to 48 and a modification site for enzyme binding. The activity $a$ was calculated for each signaling complex using Eq. (13). Binding of CheR and CheB to the complex was taken to be proportional to 1 - $a$ and $a$, respectively. Parameters for these models are given in Tables S1, S3, S6. **B1** is adapted from an analytical model presented in a previous study [4].

**Parameter values**

*1. Parameter values common to all models*

Values in Table S1 were taken from experimental measurements presented in previous studies. The basal protein counts ($R_{Tot}$, $B_{Tot}$, $T_{Tot}$) represent the mean counts per cell measured across a wild-type population by immunoblotting [3]. Parameters for the MWC model of Tar receptor clusters ($\varepsilon_0$, $\varepsilon_1$, $N$, $K$, $K^*$) were obtained through FRET measurements of kinase activity in response to doses of the chemoattractant methyl-aspartate [4]. The value of $\varepsilon_1$ reflects that in Eq. (13) $a(m, L)$ is written in terms of the mean methylation level $m$ per MWC signaling complex.

*2. Parameter values for numerical models of the receptor lattice and the analytical model with enzyme localization*

Parameter values for the adaptation kinetics were chosen to agree with recent *in vivo* measurements. All parameter values for the analytical model with enzyme localization (Table S4) are taken to agree with corresponding parameters in the numerical model **M1** (Table S2). The rates of localization of cytoplasmic CheR and CheB-P $a^t_{r,b}$ to the receptor cluster were taken from FRAP measurements [5]. We interpret these rates to represent enzymatic binding to the high-affinity tether sites. The rates of localization by binding the lower-affinity modification sites $a^m_{r,b}$ were taken to be slower. Since few enzymes localize through this channel, their exact values do not affect the predictions of the model significantly.

Key parameters for the model are $K_r$ and $K_b$ (Table S4), which characterize the affinity of tethered enzymes for the modification site and therefore the steepness of the relationship between receptor activity and the ratio of localized enzymes (Figs. 3B-D). These affinities are related to the rates in model **M1** (Table S2) via $K_{r,b} = \left(d^m_{r,b} + k_{r,b}\right)/a^m_{r*,b*}$. The values of $K_r$ and $K_b$ correspond to the Michaelis-Menten constants for the enzyme-modification site interaction divided by the effective local concentration of the tethered enzyme. Since the tether length is on the nanometer scale, these local concentrations are high: theoretical estimates based on the tether structure vary from 0.17mM [6] to 5M [7]. Given this range of estimates, we chose the values of $K_r$ and $K_b$ conservatively (*i.e.*, to be relatively large). For example, assuming Michaelis-Menten constants of 10μM, our values of $K_{r,b}$ imply an effective local concentration of 0.33mM. Smaller values of $K_{r,b}$ would lead to a stronger dependence of receptor activity on the ratio of localized enzymes and correspondingly higher predictions for the level of signaling noise.

For given values of the catalytic rates, $K_r$ and $K_b$ set the values of the binding rates of tethered enzymes to the modification sites and the rates of unbinding from the modification sites. Additionally, the values of $K_{r,b}$ constrain the values of the tether unbinding rates $d^t_{r,b}$, as discussed in the main text. Suitable choices of these rates ensure that the numbers of cytoplasmic and localized enzymes are comparable [5] and that the steady-state receptor activity is somewhat robust to variations in the expression levels of CheR and CheB. Parameters related to CheB phosphorylation were primarily based on measured values (see below).

Given values of the above parameters, the catalytic rates $k_r$ and $k_b$ of methylation and demethylation were calibrated by comparison with the measured responses of cell populations to exponential time-varying ramps of chemoattractant (Figs. 2A and S2) (Ref. [4], 32°C data). Using a CheY-CheZ FRET pair, these measurements quantified changes in receptor activity in response to ramps of methyl-aspartate. During stimulus, activity tended to reach steady-state values dependent on the speed of the ramp. These steady-state values were determined by fitting the time trace of activity to an exponential decay (Fig. S2). Our model agrees well with the experimental results over a wide range of

activity (Fig. 2A), but diverges for strong negative ramps because it does not include nonlinear CheB phosphorylation (see below).

The remaining binding rates of Table S2 were chosen to be consistent with those discussed above by requiring $a_r^t / a_r^m = a_{r*}^t / a_{r*}^m$, $a_b^t / a_b^m = a_{b*}^t / a_{b*}^m$, $a_r^t / a_b^t = a_{r*}^t / a_{b*}^t$, $a_r^m / a_b^m = a_{r*}^m / a_{b*}^m$, which satisfies detailed balance.

*3. Parameter values for models without enzyme localization*

Parameter values in Table S4 are used in the analytical model without enzyme localization and are taken from a previous study, in which they were calibrated to fit experimental measurements [4]. Parameter values (Table S5) for the numerical implementation of this model (**B1**) were derived from these values.

## CheB phosphorylation

CheB is phosphorylated by the kinase CheA. Since only phosphorylated CheB is able to efficiently dock with and demethylate chemoreceptors [5], this arrangement constitutes a negative feedback loop. We implemented a simple CheB phosphorylation loop in our numerical models (**M1**, **M2**, **M3**) and in the analytical model with enzyme localization, following previous theoretical studies [8-11]. We model CheB phosphorylation through the reaction T + CheB → T + CheB-P with rate $a_p \cdot a(\mathrm{T})$ in which $a$(T) is the activity of the receptor T. The rate of CheB-P autodephosphorylation $d_p$ has been measured by previous independent studies with excellent agreement [12,13]. We estimated the maximum phosphorylation rate $a_p$ using a simple model that considered CheA, CheB and CheY phosphorylation. Let active CheA autophosphorylate with rate $k_a$, CheA-P ($A_p$) phosphorylate CheY with rate $k_y$ = 100 μM$^{-1}$ s$^{-1}$ [14], and CheY-P ($Y_p$) dephosphorylate via CheZ with rate $k_z$ = 3 s$^{-1}$ [12]. Then at steady state

$$a k_a \left( A_{Tot} - A_p \right) \sim k_y A_p \left( Y_{Tot} - Y_p \right)$$

$$k_y A_p \left( Y_{Tot} - Y_p \right) = k_z Y_p ,$$

in which $A_{Tot}$ = 5.3 μM and $Y_{Tot}$ = 9.7 μM are the total concentrations of CheA and CheY [3] for a cell volume of 1.4 fL [15]. Solving these equations with the requirement that $Y_p$ = 2.6 μM at $a$ = 0.5 implies that $k_a \sim 3$ s$^{-1}$, which in turn implies that $A_p \sim 0.2$ μM × $a$ for most values of $a$. Since CheA-P phosphorylates CheB with rate 15 μM$^{-1}$ s$^{-1}$ [14], this estimate implies that $a_p$ = 3 s$^{-1}$ /$T_{Tot}$. In our implementation, then, the fast phosphorylation of CheA is effectively at steady state, an approximation that serves to reduce the number of parameters and significantly speed simulation.

*In vivo* measurements of activity (as measured through the CheY-CheZ interaction via FRET) suggest that the rate of CheB phosphorylation has nonlinear dependence on kinase

activity. Specifically, this dependence is inferred from: (1) significant asymmetry in the rate of the adaptation response to positive and negative step stimuli [16]; (2) a sharp increase in the demethylation rate at high kinase activities, measured through stimulation by exponential time-varying ramps of chemoattractant [4]. Comparison of results from the ramp stimulus experiments to a theoretical model indicates that CheB phosphorylation only affects the adaptation kinetics at high activities ($a > 0.74$). Since the molecular processes underlying this nonlinearity are currently unknown, we included simpler, linear CheB feedback in our models. As a result, while our model agrees well with measurements over a wide range of activities, it deviates at the highest activities (Fig. 2A). These activities are higher than the mean activities of typical, unstimulated wild-type cells, which are the primary focus of our study.

**Analytical models**

*1. Fluctuations without enzyme localization*

The model in this section is an analytical treatment of **B1**. We consider a model of MWC signaling complexes each consisting of $N = 6$ receptor dimers. Let $m$ be the average methylation level per signaling complex, varying between 0 and $8N$. We begin by studying the dynamics of the total methylation level $M = mT_{Tot}/2N$, the total number of methyl groups bound to receptors. To the equation of motion for the mean of $M$, we can apply the linear noise approximation (LNA) directly to obtain a stochastic differential equation for the dynamics of $M$. From this basis, we can then calculate fluctuations in both $m$ and the fraction of active receptor clusters $a$.

In this model we neglect the localization of the cytoplasmic enzymes with the tether site and simply assume that CheR binds the receptor modification site with affinity $(1\text{-}a)/K_r$ and CheB binds with affinity $a/K_b$. Then the number of CheR-receptor complexes is $RT/K_r$ and CheB-receptor complexes is $BT^*/K_b$, in which $R$ and $B$ are the numbers of free enzymes and $T$ and $T^*$ are the numbers of inactive and active receptors. $K_r$ and $K_b$ are Michaelis-Menten constants. Then for catalytic rates $k_r$ and $k_b$, the change in $M$ is given by $dM/dt = k_r RT/K_r - k_b BT^*/K_b$. Using the conservation of enzymes $R_{Tot} = R\left(1 + T/K_r\right)$ and $B_{Tot} = B\left(1 + T^*/K_b\right)$ yields the equation

$$\frac{dM}{dt} = \frac{k_r R_{Tot} T}{K_r + T} - \frac{k_b B_{Tot} T^*}{K_b + T^*}, \qquad \text{(S2)}$$

which has appeared in numerous previous models.

Using the LNA, we convert Eq. (S2) into the stochastic differential equation $dM = \dot{M}dt + \sqrt{D_M}\,dW$, in which $W(t)$ is the Wiener process. The relevant noise intensity is

$$D_M = \frac{k_r R_{Tot} T}{K_r + T} + \frac{k_b B_{Tot} T^*}{K_b + T^*}. \qquad \text{(S3)}$$

Since we are ultimately considering small fluctuations around the steady state, the quantities appearing in $D_M$ are evaluated at their steady-state values.

To calculate the variation in the fraction of active receptors

$$a(m) = T^*(1 + B / K_b)/T_{Tot}, \qquad \text{(S4)}$$

we apply Itō's Lemma to the function $a(m)$, yielding

$$da = \frac{\partial a}{\partial m}\frac{dM}{T_{Tot}/2N} + \frac{1}{2}\frac{\partial^2 a}{\partial m^2}\frac{D_M}{(T_{Tot}/2N)^2}dt = \frac{2N}{T_{Tot}}\frac{\partial a}{\partial m}\left[\frac{k_r R_{Tot} T}{K_r + T}dt - \frac{k_b B_{Tot} T^*}{K_b + T^*}dt + \sqrt{D_M}\,dW\right] + \frac{1}{2}\frac{\partial^2 a}{\partial m^2}\frac{D_M}{(T_{Tot}/2N)^2}dt\ . \qquad \text{(S5)}$$

The derivatives of receptor activity $a(m, L)$, Eq. (13) of the main text, with respect to the methylation level per cluster $m$ may be written in terms of the activity as

$$\frac{\partial a}{\partial m} = \varepsilon_1 a(1-a) > 0$$

$$\frac{\partial^2 a}{\partial m^2} = \varepsilon_1^2 a(1-a)(1-2a). \qquad \text{(S6)}$$

Using Eqs. (S3-6) and the conservation of receptor number

$$T_{Tot} = T\left(1 + \frac{R_{Tot}}{K_r + T}\right) + T^*\left(1 + \frac{B_{Tot}}{K_b + T^*}\right), \qquad \text{(S7)}$$

we can express $da$ in terms of only $a$ and constants. Linearizing the resulting expression around the steady state of $a$ yields an equation of the form $d(\delta a) = -(\delta a/\tau)dt + \sqrt{D_a}\,dW$ from the which the expression for the relaxation time can be read off. The variance of this process is $\mathrm{var}(\delta a) = \sigma_{aa} = \tau D_a/2$ with $D_a = (\partial a/\partial m)^2 D_M \big/ (T_{Tot}/2N)^2$. For receptor activity $a < 0.74$ and the parameters in Table S4, this model is equivalent to a recent model calibrated from population responses to exponential ramps of attractant [4]. For $a > 0.74$, that model included a nonlinear CheB-P feedback term, which we have neglected here for simplicity and since we are primarily interested in comparisons at lower activities where signaling noise is higher.

As previously discussed [8], the noise level $\sigma_{aa}$ increases with the dependence of the activity at steady state on the number of CheR, $a_0(R_{Tot})$. In Fig. S3A, $a_0(R_{Tot})$ is plotted

both for the Michaelis-Menten constants in Table S3 and reduced by a factor of 10 (Table S6). As known from Goldbeter and Koshland [17], reducing $K_r$ and $K_b$ steepens $a_0(R_{Tot})$ and correspondingly increases the noise level in activity (Fig. S3B). This model with increased affinities corresponds to the numerical model **B2**.

*2. Fluctuations in the enzyme localization model*

The dynamics of the analytical model with enzyme localization are given by the stochastic Eqs. (S8-10), adapted here from the chemical Langevin Eqs. (8-10) of the main text

$$dM = \left[\frac{k_r R^*_{Tot}(1-a)}{K_r+1-a} - \frac{k_b B^*_{p,Tot} a}{K_b+a}\right]dt + \sqrt{D_M}\, dW_M \qquad \text{(S8)}$$

$$dR^*_{Tot} = \left[a^t_r T_{Tot} R - d^t_r \frac{K_r}{K_r+1-a} R^*_{Tot}\right]dt + \sqrt{D_r}\, dW_r \qquad \text{(S9)}$$

$$dB^*_{p,Tot} = \left[a^t_b T_{Tot} B_p - d^t_b \frac{K_b}{K_b+a} B^*_{p,Tot}\right]dt + \sqrt{D_b}\, dW_b\,, \qquad \text{(S10)}$$

in which $W_i(t)$ are independent Wiener processes. The derivation is given in the main text. Additionally, enzyme numbers are conserved according to $R_{Tot} = R + R^*_{Tot}$ and $B_{Tot} = B + B_p + B^*_{p,Tot}$. Applying Itō's lemma to Eq. (S8), we find that receptor activity evolves according to

$$da = \frac{2N}{T_{Tot}}\frac{\partial a}{\partial m}\left[\frac{k_r R^*_{Tot}(1-a)}{K_r+1-a}dt - \frac{k_b B^*_{p,Tot} a}{K_b+a}dt + \sqrt{D_M}\, dW\right] + \frac{1}{2}\frac{\partial^2 a}{\partial m^2}\frac{D_M}{\left(T_{Tot}/2N\right)^2}dt\,. \qquad \text{(S11)}$$

The derivatives of activity $a$ with respect to $m$ may be expressed using Eq. (S6) as in the previous section. Equations (S9-11) may then be rewritten in the form

$$d\mathbf{X} = \mathbf{F}(\mathbf{X})dt + B d\mathbf{W}\,, \qquad \text{(S12)}$$

in which $\mathbf{X} = \left(a, R^*_{Tot}, B^*_{p,Tot}\right)^T$ and the components of the diffusion matrix $B$ are calculated by the LNA [18,19]

$$B = \text{diag}\left(\frac{2N}{T_{Tot}}\frac{\partial a}{\partial m}\left[\frac{k_r R^*_{Tot}(1-a)}{K_r+1-a} + \frac{k_b B^*_{p,Tot} a}{K_b+a}\right]^{1/2}, \left[a^t_r T_{Tot} R + d^t_r \frac{K_r}{K_r+1-a} R^*_{Tot}\right]^{1/2}, \left[a^t_b T_{Tot} B_p + d^t_b \frac{K_b}{K_b+a} B^*_{p,Tot}\right]^{1/2}\right)$$

(S13)

and evaluated at the steady state, $\mathbf{F}(\mathbf{X}_0) = 0$. To consider small deviations $\mathbf{x} = \mathbf{X} - \mathbf{X}_0$, we linearize Eq. (S12) by calculating the Jacobian $A$ of $\mathbf{F}$. The resulting linear system

$$d\mathbf{x} = A\mathbf{x}dt + Bd\mathbf{W} \qquad \text{(S14)}$$

is a multivariate Ornstein-Uhlenbeck process. The steady state variance in the output of the system is obtained by solving the Lyapunov equation [20,21]

$$A\sigma + \sigma A^T + B^T B = 0 \quad \text{(S15)}$$

for the covariance matrix $\sigma$. The autocorrelation matrix $C$ at steady state may also be calculated by

$$C(t) = \exp\left(At\right)\sigma . \qquad \text{(S16)}$$

*3. Detailed model of enzyme localization*

The analytical model of the previous section incorporates the features responsible for increased signaling noise when enzyme localization and brachiation are included in our simulation of the bacterial chemotaxis system. However, it also overestimates the magnitude of this noise. Two reasons exist for this overestimate. First, the model assumes that all receptors are equally accessible to all localized enzymes, meaning that methylation is fully distributive. In this picture, enzymes bind the receptors for which they have the highest affinity (low methylation level for CheR and high methylation level for CheB) regardless of the state of the receptor to which they are tethered. The result is an overestimate of the binding affinity of localized enzymes for the receptor substrate. Second, the previous analytical model does not consider a distribution of methylation levels. Rather, $\partial a / \partial m$ is evaluated at only a single methylation level corresponding to the mean activity of the system. This approximation tends to overestimate $\partial a / \partial m$, most significantly for systems with mean activity of $a \sim 0.5$, where $\partial a / \partial m$ is largest. Addressing these issues requires a model that considers the dynamics of MWC complexes of each methylation level $m$ individually. Additionally, the model must track the numbers of enzymes localized to complexes at each methylation level. To tune the processivity of methylation, we introduce a parameter $\beta$ representing the rate at which localized enzymes randomize their position. The value $\beta = 0$ corresponds to completely processive methylation and the limit $\beta \to \infty$ corresponds to purely distributive methylation, reducing the model to Eqs. (8-10) of the main text.

Let $T_{Tot,\,m}$ be the total number of receptor monomers within MWC signaling complexes of methylation level $m$. Also let $R_m^*$ and $B_{p,m}^*$ be the number of CheR and CheB-P localized within clusters of methylation level $m$ but not bound to modification sites. For simplicity, we consider only the case of a tethered enzyme binding a modification site in the same cluster that it is localized. This binding of the modification site forms complexes denoted by $\overline{R_m^* T_m}$ and $\overline{B_{p,m}^* T_m}$ . The changes in the number of these complexes due to modification site (un)binding and catalysis is then:

$$\frac{d}{dt}R_m^*T_m = a_{r*}^m\left(1-a_m\right)R_m^* - \left(d_r^m + k_r\right)\overline{R_m^*T_m}$$

$$\frac{d}{dt}B_{p,m}^*T_m = a_{b*}^m a_m B_{p,m}^* - \left(d_b^m + k_b\right)\overline{B_{p,m}^*T_m} \qquad \text{(S17)}$$

Taking the approximation $dR_m^*T_m/dt = dB_{p,m}^*T_m/dt = 0$ as in Eqs. (5-7) of the main text, changes in $T_{Tot,\,m}$, are given by

$$\frac{d}{dt}T_{Tot,0} = -\frac{k_r}{K_r}R_0^*\left(1-a_0\right) + \frac{k_b}{K_b}B_{p,1}^*a_1$$

$$\vdots$$

$$\frac{d}{dt}T_{Tot,m} = \frac{k_r}{K_r}R_{m-1}^*\left(1-a_{m-1}\right) - \frac{k_r}{K_r}R_m^*\left(1-a_m\right) - \frac{k_b}{K_b}B_{p,m}^*a_m + \frac{k_b}{K_b}B_{p,m+1}^*a_{m+1} \qquad \text{(S18)}$$

$$\vdots$$

$$\frac{d}{dt}T_{Tot,8N} = \frac{k_r}{K_r}R_{8N-1}^*\left(1-a_{8N-1}\right) - \frac{k_b}{K_b}B_{p,8N}^*a_{8N}$$

in which the cluster activity $a_m = a(m, L)$. All parameters are defined in the same manner as those for the previous analytical model given in Table S4.

We now write the analogs of Eqs. (1, 2) in the main text, which here describe the binding and unbinding of cytoplasmic CheR ($R$) and CheB-P ($B_p$) to tether sites within signaling complexes of methylation level $m$. The quantities

$$R_{Tot,m}^* = R_m^*\left(1 + \frac{1-a_m}{K_r}\right),$$

$$B_{p,Tot,m}^* = B_{p,m}^*\left(1 + \frac{a_m}{K_b}\right) \qquad \text{(S19)}$$

denote the total numbers of localized enzymes at each methylation level. These total localized enzyme counts evolve according to

$$\frac{d}{dt}R_{Tot,m}^* = a_r^t R T_{Tot,m} - d_r^t R_m^* + \frac{k_r}{K_r}\left[R_{m-1}^*\left(1-a_{m-1}\right) - R_m^*\left(1-a_m\right)\right] + \beta\left(-R_m^* + R^*\frac{T_{Tot,m}}{T_{Tot}}\right)$$

$$\text{(S20)}$$

$$\frac{d}{dt}B^*_{p,Tot,m} = a^t_b B_p T_{Tot,m} - d^t_b B^*_{p,m} + \frac{k_b}{K_b}\left(-B^*_{p,m}a_m + B^*_{p,m+1}a_{m+1}\right) + \beta\left(-B^*_{p,m} + B^*_p \frac{T_{Tot,m}}{T_{Tot}}\right)$$

(S21).

The first term two terms of the right hand side arise from the binding and unbinding of localized enzymes from the tether site. As in the previous model, we here assume the number of free tethers to be large compared to the number of localized enzymes and therefore $\sim T_{Tot,m}$. The third and fourth terms (multiplied by $k_{r,b}/K_{r,b}$) represent the localized enzyme (de)methylating the complex within which it is localized. The last two terms introduce a redistribution of localized enzymes between the receptor clusters occurring at a rate $\beta$. The probability of a given complex receiving a new enzyme is proportional to its relative abundance $T_{Tot,m}/T_{Tot}$.

Molecule counts are conserved according to

$$R_{Tot} = R + \sum_m R^*_{Tot,m}$$

$$B_{Tot} = B + B_p + \sum_m B^*_{p,Tot,m} \qquad \text{(S22)}$$

$$T_{Tot} = \sum_m T_{Tot,m}$$

and the overall activity $a$ is calculated according to

$$a = \frac{1}{T_{Tot}}\sum_m a_m T_{Tot,m} . \qquad \text{(S23)}$$

In the limit $\beta \to \infty$, Eqs. (S18, S20, S21) reduce to Eqs. (8-10) of the main text. Summing Eqs. (S20, S21) over $m$ and using the definitions $R^* = \sum_m R^*_m$ and $B^*_p = \sum_m B^*_{p,m}$ reduces them to Eqs. (9, 10) describing the dynamics of the total number of localized enzymes. To recover Eq. (8), we note that as $\beta \to \infty$, it follows from Eqs. (S20, S21) that $R^*_m \sim R^* T_{Tot,m}/T_{Tot}$ and $B^*_{p,m} \sim B^*_p T_{Tot,m}/T_{Tot}$. Inserting these values into Eq. (S18) and summing $dm/dt \sim \sum_m m\dot{T}_{Tot,m}/T_{Tot}$ yields Eq. (8) for $a(m = 0) \sim 0$ and $a(m = 8N) \sim 1$ [8]. This limit corresponds to fully distributive methylation in which localized enzymes are completely redistributed between each methylation event according to the abundances $T_{Tot,m}$.

For calculations about the steady state with no stimulus, only methylation levels up to $m \sim 16$ must be considered, since $a(16, L = 0) \sim 1$ guarantees that CheR will not methylate clusters beyond this limit. Signaling noise in the overall activity $a$ is calculated by

linearizing and applying the linear noise approximation to Eqs. (S18, S20, S21). The variance in the overall activity $\sigma_{aa}$ is calculated from the covariances of the $T_{Tot,m}$. Results of this calculation for $\beta = 0$ (fully processive) and $\beta = 20\ s^{-1}$ (more distributive) are shown in Fig. S4. The more distributive model exhibits larger fluctuations and a higher affinity of the localized enzymes for the receptor substrate.

**Comparing the dependence of receptor activity on the localized enzyme ratio**

When calculating the localized CheR to CheB-P ratio for the numerical models (Fig. 4C, D), we ignore the population of "inert" CheR and CheB-P localized respectively within fully methylated or demethylated assistance neighborhoods or, for the model **M2** lacking assistance neighborhoods, tethered to fully methylated or demethylated receptor dimers. While these inert enzymes are able to bind the modification sites of the receptors, they are unable to participate in methylation-demethylation and therefore do not affect the activity of the receptor cluster. This consideration provides the best comparison to Fig. 4B, since the analytical model assumes all bound enzymes will (de)methylate receptors at the same rate, Eq. (7). The population of inert CheR is small for all models, but the fraction of inert CheB is high for the processive models since many receptors are fully demethylated (Fig. S6). We note that since the simulated MWC complexes in the absence of stimulus are half active when the methylation of the complex is $m = 6$ (out of a possible 48) and almost fully active with $m = 12$, fully demethylated receptors are common even when the activity of the receptor cluster is high.

**Comparing adaptation rates between the numerical models**

When presented with a large attractant stimulus, the numerical models clearly displayed different rates of adaptation with the most distributive model **M1** reaching its adapted level of activity first (Fig. 2B). Fig. S7 shows the mean methylation level per MWC complex versus time for the simulations plotted in Fig. 2B, which clearly shows that **M1** (black) has the highest overall methylation rate during adaptation. We note that since the enzymes in model **M3** have lower rates of tether unbinding, ~30% more enzymes are localized to the cluster than in **M1**. This leads to the slightly faster initial rate of methylation for **M3** compared to **M1**. This rate, however, drops due to the processivity of methylation in **M3**, as localized CheR is unable to escape from pockets of high methylation. In contrast, the methylation rate of the most distributive model **M1** remains nearly constant during adaptation.

**References**


1. Sneddon MW, Faeder JR, Emonet T (2011) Efficient modeling, simulation and coarse-graining of biological complexity with NFsim. Nat Methods 8: 177-183.
2. Faeder JR, Blinov ML, Hlavacek WS (2009) Rule-based modeling of biochemical systems with BioNetGen. Methods Mol Biol 500: 113-167.
3. Li MS, Hazelbauer GL (2004) Cellular stoichiometry of the components of the chemotaxis signaling complex. J Bacteriol 186: 3687-3694.
4. Shimizu TS, Tu YH, Berg HC (2010) A modular gradient-sensing network for chemotaxis in Escherichia coli revealed by responses to time-varying stimuli. Mol Syst Biol 6: 382.
5. Schulmeister S, Ruttorf M, Thiem S, Kentner D, Lebiedz D, et al. (2008) Protein exchange dynamics at chemoreceptor clusters in Escherichia coli. P Natl Acad Sci USA 105: 6403-6408.
6. Levin MD, Shimizu TS, Bray D (2002) Binding and diffusion of CheR molecules within a cluster of membrane receptors. Biophys J 82: 1809-1817.
7. Windisch B, Bray D, Duke T (2006) Balls and chains - A mesoscopic approach to tethered protein domains. Biophys J 91: 2383-2392.
8. Emonet T, Cluzel P (2008) Relationship between cellular response and behavioral variability in bacterial chernotaxis. P Natl Acad Sci USA 105: 3304-3309.
9. Kollmann M, Lovdok L, Bartholome K, Timmer J, Sourjik V (2005) Design principles of a bacterial signalling network. Nature 438: 504-507.
10. Rao CV, Kirby JR, Arkin AP (2004) Design and diversity in bacterial chemotaxis: A comparative study in Escherichia coli and Bacillus subtilis. Plos Biol 2: 239-252.
11. Vladimirov N, Lovdok L, Lebiedz D, Sourjik V (2008) Dependence of bacterial chemotaxis on gradient shape and adaptation rate. Plos Comput Biol 4: e1000242.
12. Kentner D, Sourjik V (2009) Dynamic map of protein interactions in the Escherichia coli chemotaxis pathway. Mol Syst Biol 5: 238.
13. Stewart RC (1993) Activating and inhibitory mutations in the regulatory domain of CheB, the methylesterase in bacterial chemotaxis. The Journal of biological chemistry 268: 1921-1930.
14. Stewart RC, Jahreis K, Parkinson JS (2000) Rapid phosphotransfer to CheY from a CheA protein lacking the CheY-binding domain. Biochemistry 39: 13157-13165.
15. Sourjik V, Berg HC (2002) Binding of the Escherichia coli response regulator CheY to its target measured in vivo by fluorescence resonance energy transfer. P Natl Acad Sci USA 99: 12669-12674.
16. Sourjik V, Berg HC (2002) Receptor sensitivity in bacterial chemotaxis. P Natl Acad Sci USA 99: 123-127.
17. Goldbeter A, Koshland DE (1981) An Amplified Sensitivity Arising from Covalent Modification in Biological-Systems. P Natl Acad Sci-Biol 78: 6840-6844.
18. Gillespie DT (2000) The chemical Langevin equation. J Chem Phys 113: 297-306.
19. Elf J, Ehrenberg M (2003) Fast evaluation of fluctuations in biochemical networks with the linear noise approximation. Genome Res 13: 2475-2484.
20. Gardiner CW (2004) Handbook of stochastic methods for physics, chemistry, and the natural sciences. Berlin; New York: Springer-Verlag. 415 p.
21. Kampen NGv (2007) Stochastic processes in physics and chemistry. Amsterdam; New York: Elsevier. 463 p.
